# Association between adiposity levels and cognitive impairment in the Chilean older adult population

**DOI:** 10.1017/jns.2019.24

**Published:** 2019-10-09

**Authors:** Y. Concha-Cisternas, F. Lanuza, H. Waddell, Anne Sillars, A. M. Leiva, C. Troncoso, M. A. Martinez, M. Villagrán, L. Mardones, M. Martorell, G. Nazar, N. Ulloa, A. M. Labraña, X. Diaz-Martinez, K. Sadarangani, C. Alvarez, R. Ramirez-Campillo, Alex Garrido-Mendez, Cristian Luarte, Frederick Ho, Stuart R. Gray, F. Petermann-Rocha, C. Celis-Morales

**Affiliations:** 1 Escuela de Kinesiología, Facultad de Salud, Universidad Santo Tomás, Talca, Chile; 2 Universidad Autónoma de Chile, Talca, Chile; 3 Departamento de Pediatría y Cirugía Infantil, Universidad de la Frontera, Temuco, Chile; 4 Institute of Cardiovascular and Medical Science, University of Glasgow, Glasgow, UK; 5 Instituto de Anatomía, Histología y Patología, Facultad de Medicina, Universidad Austral de Chile, Valdivia, Chile; 6 CIEDE-UCSC, Facultad de Medicina, Universidad Católica de la Santísima Concepción, Concepción, Chile; 7 Instituto de Farmacia, Facultad de Ciencias, Universidad Austral de Chile, Valdivia, Chile; 8 Departamento de Ciencias Básicas, Universidad Católica de la Santísima Concepción, Concepción, Chile; 9 Departamento de Nutrición y Dietética, Facultad de Farmacia, Universidad de Concepción, Concepción, Chile; 10 Departamento de Psicología y Centro de Vida Saludable, Universidad de Concepción, Concepción, Chile; 11 Departamento de Bioquímica Clínica e Inmunología, Facultad de Farmacia y Centro de Vida Saludable de la Universidad de Concepción, Concepción, Chile; 12 Grupo de Investigación Calidad de Vida, Departamento Ciencias de la Educación, Universidad del Biobío, Chillán, Chile; 13 Escuela de Kinesiología, Facultad de Ciencias de la Salud, Universidad San Sebastián, Santiago, Chile; 14 Escuela de Kinesiología, Facultad de Salud y Odontología, Universidad Diego Portales, Santiago, Chile; 15 Grupo de Investigación en Área Prioritaria Bienestar Humano y Calidad de Vida, Departamento de Ciencias de la Actividad Física, Actividad Física y Deporte, Universidad de los Lagos, Osorno, Chile; 16 Departamento de Ciencias del Deporte y Acondicionamiento Físico, Universidad Católica de la Santísima Concepción, Concepción, Chile; 17 Escuela de Educación Física, Universidad San Sebastián, Concepción, Chile; 18 Institute of Health and Wellbeing, University of Glasgow, Glasgow, UK; 19 Centro de Investigación en Fisiología del Ejercicio (CIFE), Universidad Mayor, Santiago, Chile

**Keywords:** Cognitive impairment, Adiposity, Obesity, Ageing, Elderly, CNHS, Chilean National Health Survey, MMSE, Mini-Mental State Examination, WC, waist circumference

## Abstract

Although both obesity and ageing are risk factors for cognitive impairment, there is no evidence in Chile on how obesity levels are associated with cognitive function. Therefore, the aim of the present study was to investigate the association between adiposity levels and cognitive impairment in older Chilean adults. This cross-sectional study includes 1384 participants, over 60 years of age, from the Chilean National Health Survey 2009–2010. Cognitive impairment was evaluated using the Mini-Mental State Examination. BMI and waist circumference (WC) were used as measures of adiposity. Compared with people with a normal BMI, the odds of cognitive impairment were higher in participants who were underweight (OR 4·44; 95 % CI 2·43, 6·45; *P* < 0·0001), overweight (OR 1·86; 95 % CI 1·06, 2·66; *P* = 0·031) and obese (OR 2·26; 95 % CI 1·31, 3·21; *P* = 0·003). The associations were robust after adjustment for confounding variables. Similar results were observed for WC. Low and high levels of adiposity are associated with an increased likelihood of cognitive impairment in older adults in Chile.

In recent decades there has been a demographic shift characterised by an increase in the proportion of the population over 60 years of age^(1)^. Indeed, it has been estimated that between 2015 and 2050 this population will rise to more than two billion people^(2)^. This shift has been observed in the Chilean population, where the prevalence of older adults is expected to increase from 19·9 % in 2017 to 21·6 % in 2050, reaching an average age of 80·5 years^(3)^. This will make it the nation with the highest life expectancy in Latin America^(3)^ and thus susceptible to the concurrent problems associated with an ageing population. Indeed ageing is, unfortunately, accompanied by a marked increase in chronic non-communicable diseases such as diabetes mellitus type 2, arterial hypertension and some types of cancer^(4)^.

On top of this, in Chile, an increase in the prevalence of underweight and obesity has been reported, affecting 7·8 and 65·4 % of people aged over 65 years, respectively^(5)^. Older adults are likely to experience important body composition changes inherent with ageing^(6)^; however, in many cases, this undernutrition or obesity may be related to the adoption of unhealthy lifestyles, including poor diet and low levels of physical activity^(7,8)^. Previous studies have shown that underweight and obesity cause functional limitation^(9)^ and disability^(10)^ in the older adult. It has also been associated with some geriatric syndromes, such as urinary incontinence, falls and cognitive impairment^(11–14)^. Furthermore, cognitive impairment has been ranked as the sixth leading cause of death, and is responsible for 4 % of premature deaths in adults aged >60 years, in Chile^(15)^.

As a result, nutritional status must be assessed as a health indicator in older adults, where BMI and waist circumference (WC), considered validated measurements of adiposity and metabolic risk, may be associated with the development of neurodegenerative diseases^(16,17)^. Several studies have suggested that having high adiposity and, therefore, a high BMI and WC during adulthood, increases the probability of developing cognitive impairment and dementia in old age^(12,18,19)^. In contrast with this, some studies indicate that older adults with low, but not high, BMI are at increased risk of dementia, going so far as to suggest that high adiposity could be protective^(20–22)^. Such associations have yet to be investigated in Chile. Therefore, the aim of the present study was to investigate the association between adiposity and cognitive impairment in the older adult population in Chile.

## Materials and methods

### Study design

This cross-sectional study included a total of 1384 adults aged over 60 years (based on the WHO definition of older/elderly persons) from the 2009 to 2010 Chilean National Health Survey (CNHS)^(23)^. The CNHS is a cross-sectional study conducted in homes from a national, probabilistic, stratified and multistage sample and includes people with an age range of 15−98 years with national, regional and urban/rural representativeness. The present study was conducted according to the guidelines laid down in the Declaration of Helsinki and all procedures involving human subjects/patients were approved by the Ethics Committee of the School of Medicine at the Pontifical Catholic University of Chile (approval no. 09–113). Written informed consent was obtained from all participants^(23)^.

### Abbreviated Mini-Mental State Examination

The abbreviated version of the Mini-Mental State Examination (MMSE) was used to measure cognitive impairment^(23)^. This instrument has been widely used due to its proven high reliability and has been validated previously in the Chilean population^(24,25)^. It evaluates executive function, visuo-constructive capacity, memory, ability to understand instructions and temporo-spatial orientation^(26)^. The abbreviated version consists of six questions, with a maximum score of nineteen points. A score below 13 on the MMSE indicates the presence of cognitive impairment^(23)^. This abbreviated version has showed a higher sensitivity and specificity than the original long-format version^(27)^.

### Determination of adiposity levels

Body weight, height and WC were measured by trained nurses using standardised protocols. BMI was derived from body weight divided by height squared and nutritional status was derived using the following cut-off points: underweight: ≤18·5 kg/m^2^; normal weight: 18·5–24·9 kg/m^2^; overweight: 25·0–29·9 kg/m^2^ and obesity: ≥30·0 kg/m^2(28)^. Central obesity was defined as a WC ≥88 cm for women and ≥102 cm for men^(23)^. In order to assess the association between cognitive impairment and WC, sex-specific quartiles were derived for men (quartile 1: <89 cm; quartile 2: 89–97 cm; quartile 3: 98–104 cm; quartile 4: >104 cm) and women (quartile 1: <86 cm; quartile 2: 86–93 cm; quartile 3: 94–102 cm; quartile 4: >102 cm).

### Sociodemographic and lifestyle variables

Sociodemographic variables such as age, sex, education level (elementary <8 years, secondary: 8−12 years, higher education >12 years), income (low <250 000; medium: 250 000 to 650 000; high >650 000 Chilean pesos) and lifestyle factors such as smoking, drinking alcohol, and fruit and vegetable intake data were obtained by validated questionnaires, as described previously^(23)^.

The total level of physical activity was determined using the Global Physical Activity Questionnaire (GPAQ v2)^(29)^, which has previously been validated in the Latino population^(30)^. Total physical activity is presented as the sum of the time reported in transport-related physical activity, moderate and vigorous intensity physical activity. This variable was expressed in metabolic equivalents (MET) for min/week. The cut-off for physical inactivity was an energy output of less than 600 MET-min/week^(29,31)^. The levels of sedentary behaviour were determined using the same questionnaire – GPAQ v2 – from the self-report of time devoted to activities that involve sitting or reclining in free time or at work (e.g. time seated in front of the computer or television, travelling by bus, train or car, amongst others). A person who spent more than 4 h per d sitting (equivalent to the population median) was considered to have a high level of sedentary behaviour^(32)^.

In order to assess participant lifestyle, seven lifestyle factors were examined and scored to produce a healthy lifestyle score. The seven lifestyle factors were as follows: consuming five portions of fruit and vegetables per d, consuming <8 g of salt per d, an audit alcohol score of <8 (units), sleeping between 7 and 9 h per d, having never smoked, physical activity (>150 min a week of moderate-to-vigorous physical activity) and time spent sitting <4 h per d. Participants were assigned one point for each healthy behaviour and those who did not meet these recommendations were assigned a value of 0. The score was then categorised as unhealthy (≤1 healthy behaviours), slightly healthy (2–3 healthy behaviours), healthy (4–5 healthy behaviours) and very healthy (>5 healthy behaviours).

### Statistical analyses

The general characteristics of the study population are presented as means and standard deviations for continuous variables, and as percentages for categorical variables. In order to investigate the association between adiposity levels (BMI and WC) and cognitive impairment, logistic regression analysis was performed, and the results presented as the OR and 95 % CI. Participants with a normal BMI were defined as the reference group, whereas for WC the reference group were subjects in quartile 2 (86–93 cm for women and 89–97 cm for men). All analyses were adjusted for confounding variables using four statistical models: model 0 – unadjusted; model 1 – adjusted for age, sex, region and residency area (urban and rural); model 2 additionally adjusted for education level and income status; model 3 additionally adjusted for healthy lifestyle score; model 4 was adjusted form model 3 but it was also mutually adjusted for BMI and WC in order to identify whether the association of cognitive impairment with overall adiposity (BMI) was independent of central adiposity measured (WC) and vice versa. For all analyses the complex samples module in STATA SE v14 was used. The significance level was defined as *P* < 0·05.

### Ethical standards disclosure

The CNHS 2009–2010 was funded by the Chilean Ministry of Health and led by the Department of Public Health, The Pontificia Universidad Católica de Chile. The CNHS 2009–2010 was conducted according to the guidelines laid down in the Declaration of Helsinki and all procedures involving human subjects were approved by the Ethics Research Committee of the Faculty of Medicine at the Pontificia Universidad Católica of Chile. Written informed consent was obtained from all subjects.

## Results

The characteristics of the study population according to the results of the normal and altered MMSE are shown in Table 1. In the descriptive data for sociodemographic variables, it can be noted that participants with cognitive impairment were older, and had lower education level and income. With respect to anthropometric measurements, participants with cognitive impairment were more likely to be at the extremes of BMI (underweight, overweight and obesity); however, WC was not different in normal individuals compared with those with cognitive impairment (99·8 *v.* 98·3 cm, respectively). In relation to lifestyle, an increased prevalence of physical inactivity, a greater number of time spent sitting during the day, and worse self-reported health and well-being was observed in participants with cognitive impairment.

**Table 1.** Population characteristics according to the Mini-Mental State Examination (Numbers of subjects; percentages and 95 % confidence intervals; mean values and 95 % confidence intervals)

	No cognitive impairment		Cognitive impairment	
	%	95 % CI	%	95 % CI
Sociodemographic				
Subjects (*n*)	1215		169	
Prevalence	88·4	84·8, 91·2	11·6	8·8, 15·2
Sex				
Men	44·4	39·1, 49·8	52·6	38·6, 66·2
Women	55·6	50·2, 60·9	47·3	33·8, 61·4
Age (years)				
Mean	68·5		76·3	
95 % CI	67·8, 69·2		73·8, 78·8	
Area of residency				
Rural	14·4	11·8, 17·5	29·5	16·4, 47·1
Urban	85·6	82·4, 88·2	70·5	52·8, 83·5
Education level				
Elementary	47·0	41·8, 52·0	84·4	73·5, 91·3
Secondary	38·0	32·8, 43·4	14·4	7·8, 25·2
Technical university	15·0	10·9, 20·3	1·2	0·2, 4·7
Income level				
Low	56·5	51·1, 61·7	83·5	71·2, 91·1
Middle	32·6	27·7, 37·9	16·5	8·8, 28·7
High	10·9	7·5, 15·6	0	
Anthropometrics				
Height (m)				
Mean	1·58		1·54	
95 % CI	1·57, 1·59		1·53, 1·57	
Body weight (kg)				
Mean	71·6		65·2	
95 % CI	70·2, 72·9		61·9, 68·6	
BMI (kg/m^2^)				
Mean	28·7		27·2	
95 % CI	28·1, 29·3		25·9, 28·6	
Nutritional status				
Underweight (<18·5 kg/m^2^)	1·1	0·4, 2·4	2·1	0·5, 7·3
Normal (18·5–24·9 kg/m^2^)	24·0	19·2, 29·3	34·5	22·2, 49·1
Overweight (25·0–29·9 kg/m^2^)	41·7	36·6, 46·9	34·3	21·2, 47·3
Obesity (≥30·0 kg/m^2^)	33·3	28·6, 38·4	29·2	19·6, 41·1
Waist circumference (cm)	95·7	94·6, 96·9	93·5	90·2, 96·9
Central obesity				
Normal	47·3	42·1, 52·5	44·0	30·8, 58·1
Obese	52·7	47·4, 57·9	56·0	41·9, 69·2
Waist circumference quartiles				
Lowest	26·8	22·4, 31·8	35·0	23·1, 49·0
Middle-low	24·2	19·8, 29·3	21·9	13·1, 34·3
Middle-high	25·2	20·8, 30·2	17·4	10·3, 27·7
Highest	23·5	19·4, 28·2	25·6	16·8, 37·0
Lifestyle				
Total physical activity (MET/h per week)				
Mean	90·3		47·1	
95 % CI	76·7, 103·8		22·2, 71·9	
Prevalence of physical inactivity				
Active	69·9	65·1, 74·3	36·6	24·7, 50·4
Inactive	30·1	25·7, 34·9	63·4	49·6, 75·3
Sitting time (h/d)				
Mean	3·67		5·46	
95 % CI	3·30, 4·04		3·84, 7·07	
Fruit and vegetables intake (g/d)				
Mean	243·0		196·3	
95 % CI	226·6, 259·4		165·7, 227·0	
Alcohol intake (g/d)				
Mean	39·5		42·5	
95 % CI	29·4, 49·6		24·6, 60·5	
Hours of sleep				
<7 h	49·4	44·1, 54·6	32·0	20·3, 46·4
7–9 h	34·5	29·6, 39·8	24·8	16·2, 36·1
>9 h	16·1	12·9, 19·8	43·1	29·2, 58·2
Healthy lifestyle score				
Unhealthy	30·3	35·1, 36·1	15·3	8·2, 26·5
Slightly healthy	26·6	21·7, 32·2	23·9	14·6, 36·6
Healthy	22·8	18·4, 27·8	28·9	15·5, 47·3
Very healthy	20·3	16·6, 24·5	31·8	15·8, 53·7
Smoking				
Never	45·4	40·2, 50·5	54·0	39·1, 68·2
Ex-smoker	34·9	29·8, 40·2	33·4	24·2, 54·9
Smoker	19·8	15·8, 24·3	7·6	3·5, 15·5
Self-reported health and well-being				
Poor	2·8	1·5, 5·0	9·9	5·4, 17·5
Average	34·2	29·4, 39·2	57·1	43·2, 70·0
Good	63·0	57·9, 67·8	32·9	21·7, 46·5
Mini-mental score				
Mean	17·1		8·6	
95 % CI	16·9, 17·3		7·6, 9·5	

MET, metabolic equivalents.

**Fig. 1.**
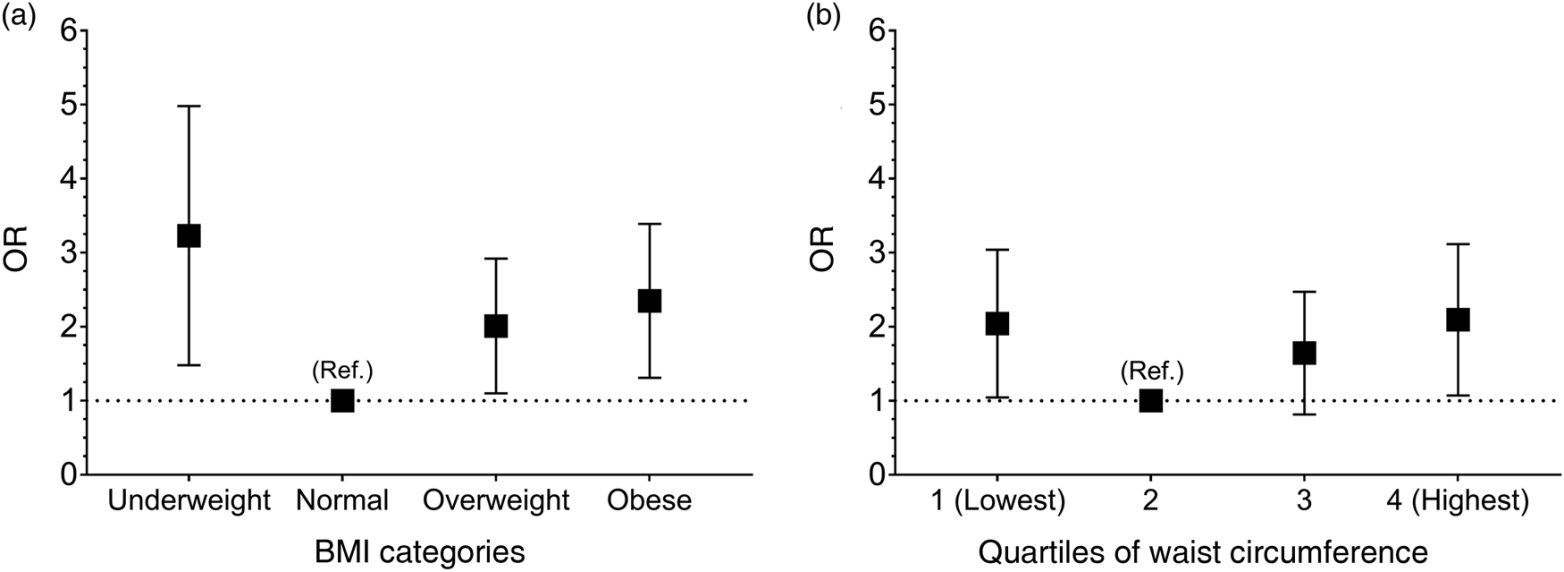
Risk for cognitive impairment according to BMI (a) or waist circumference (b). Data are presented as odds ratios and their respective 95 % confidence intervals, represented by vertical bars. Ref.: the baseline group was comprised of people with normal BMI according to classification in older adults or waist circumference. A value greater than 1 indicates an increased probability of cognitive impairment (Mini-Mental State Examination <13). The analyses were adjusted for age, sex, region, geographical area, education level, socio-economic level and healthy lifestyle points. The cut-off for the waist circumference quartiles were sex-specific (men quartile 1: <89 cm; quartile 2: 89–97 cm; quartile 3: 98–104 cm; quartile 4: >104 cm; and women quartile 1: <86 cm; quartile 2: 86–93 cm; quartile 3: 94–102 cm; quartile 4: >102 cm). The cut-off points for BMI were: underweight <18·5 kg/m^2^; normal weight: 18·5–24·9 kg/m^2^; overweight: 25·0–29·9 kg/m^2^ and obesity: ≥30·0 kg/m^2^.

The association between BMI and cognitive impairment is shown in Table 2. The statistically unadjusted model, model 0, identified an increase in the likelihood of cognitive impairment in those who were underweight (OR 4·44; 95 % CI 2·43, 6·45; *P* < 0·0001), overweight (OR 1·86; 95 % CI 1·06, 2·66; *P* = 0·031) and obese (OR 2·26; 95 % CI 1·31, 3·21; *P* = 0·003) when compared with those with a normal BMI. Models 2, 3 and 4 were adjusted for other confounding factors which reduced the magnitude of the resulting associations; however, the associations remained significant (Table 2 and Fig. 1(a)). When the association between BMI and cognitive impairment was adjusted for WC, the associations were attenuated but remained significant for underweight (OR 3·00; 95 % CI 1·48, 4·52; *P* = 0·002) and obese (OR 2·13; 95 % CI 1·12, 3·14; *P* = 0·021) individuals (Table 2).

**Table 2.** Association between BMI levels and cognitive impairment (Odds ratios and 95 % confidence intervals)

	Underweight (<18·5 kg/m^2^)			Normal (18·5–24·9 kg/m^2^)		Overweight (25·0–29·9 kg/m^2^)			Obese (≥30·0 kg/m^2^)		
Models	OR	95 % CI	*P*	OR	95 % CI	OR	95 % CI	*P*	OR	95 % CI	*P*
Model 0	4·44	2·43, 8·12	<0·0001	1·00	Ref.*	1·86	1·06, 3·29	0·031	2·26	1·31, 3·90	0·003
Model 1	3·35	1·76, 6·39	<0·0001	1·00	Ref.	2·06	1·14, 3·72	0·016	2·46	1·38, 4·36	0·002
Model 2	3·24	1·69, 6·22	<0·0001	1·00	Ref.	2·01	1·11, 3·65	0·022	2·35	1·32, 4·21	0·004
Model 3	3·23	1·68, 6·20	<0·0001	1·00	Ref.	2·01	1·10, 3·64	0·022	2·35	1·31, 4·20	0·004
Model 4	3·00	1·48, 6·06	0·002	1·00	Ref.	1·95	0·87, 4·38	0·102	2·13	1·12, 4·07	0·021

Ref., reference; MMSE, Mini-Mental State Examination.

* Ref.: the baseline group was comprised of people with normal BMI for older adults. A value greater than 1 indicates an increased risk of cognitive impairment (MMSE <13). The models were constructed as follows: model 0 – not adjusted; model 1 – adjusted for age, sex, region and geographical area; model 2 was adjusted for model 1, but also for education level and socio-economic level; model 3 was adjusted for model 2, but also for healthy lifestyle points; model 4 was adjusted for model 3 but also for waist circumference.

The association between WC levels and cognitive impairment are presented in Table 3. Participants in the lowest WC quartile, quartile 1, had a greater likelihood of cognitive impairment (OR 2·20; 95 % CI 1·17, 4·16; *P* = 0·014) compared with participants in quartile 2. Additionally, similar results were observed in participants who were in the highest compared with the lowest WC quartile (OR 2·21; 95 % CI 1·16, 4·19; *P* = 0·015). However, participants in quartile 3 did not show a significant association with cognitive impairment. When these analyses were adjusted for confounding variables, the associations were weaker but remained statistically significant (Table 3 and Fig. 1(b)). When the association between WC and cognitive impairment was adjusted for BMI, the associations were only observed for the highest quartile, which somehow shows a stronger magnitude of association when BMI was included as a confounding factor in the model (Table 3).

**Table 3.** Association between waist circumference and cognitive impairment (Odds ratios and 95 % confidence intervals)

	Quartile 1 (lowest)			Quartile 2		Quartile 3			Quartile 4 (highest)		
Models	OR	95 % CI	*P*	OR	95 % CI	OR	95 % CI	*P*	OR	95 % CI	*P*
Model 0	2·20	1·17, 4·16	0·014	1·00	Ref.*	1·77	0·91, 3·45	0·092	2·21	1·16, 4·19	0·015
Model 1	1·83	0·94, 3·53	0·071	1·00	Ref.	1·46	0·73, 2·93	0·275	2·03	1·05, 3·95	0·035
Model 2	2·06	1·05, 4·01	0·033	1·00	Ref.	1·64	0·81, 2·31	0·163	2·12	1·08, 3·14	0·028
Model 3	2·04	1·05, 3·03	0·036	1·00	Ref.	1·64	0·81, 2·47	0·164	2·09	1·07, 3·11	0·031
Model 4	1·37	0·61, 3·04	0·436	1·00	Ref.	1·32	0·62, 2·78	0·465	3·06	1·47, 6·38	0·003

Ref., reference; MMSE, Mini-Mental State Examination.

* Ref.: the baseline group was comprised of people with a waist circumference classified in quartile 2. A value greater than 1 indicates an increased risk of cognitive impairment (MMSE <13). The models were constructed as follows: model 0 – not adjusted; model 1 – adjusted for age, sex, region and geographical area; model 2 was adjusted for model 1, but also for education level and socio-economic level; model 3 was adjusted for model 2, but also for healthy lifestyle points; model 4 was adjusted for model 3 but also for BMI. The cut-offs for the waist circumference quartiles were sex-specific (men quartile 1: <89 cm; quartile 2: 89–97 cm; quartile 3: 98–104 cm; quartile 4: >104 cm; and women quartile 1: <86 cm; quartile 2: 86–93 cm; quartile 3: 94–102 cm; quartile 4: >102 cm).

## Discussion

The results of the present study confirm that both high and low levels of adiposity, quantified through BMI and WC, were associated with a higher probability of cognitive impairment in the older adult population. The findings were robust after adjusting for confounding variables, including sociodemographic and lifestyle factors. In particular, older people who were underweight appeared to be more likely to have cognitive impairment than those who were overweight or obese. These results have the potential to inform the development of programmes or interventions for older adults in Chile and other nations in Latin America, including better lifestyle guidance for appropriate weight maintenance, and cognitive screening for both overweight and underweight older people. Considering the large numbers of older adults in Chile and the projection for this population to grow over the next three decades, the implications of these programmes could have a large social and economic impact.

The findings of the present study are largely consistent with the existing literature. For example, a prospective cohort study has found that BMI and WC were independent risk factors for dementia, where the presence of high BMI (>30·0 kg/m^2^) and higher WC were associated with a 3·6-fold risk, compared with those only with normal BMI and lower WC^(18)^. Additionally, longitudinal studies have revealed that a high BMI is a risk factor for cognitive impairment^(33,34)^, and a recent meta-analysis indicated that obesity in middle age is a risk factor that increases the probability of developing dementia in old age^(35)^. The association between obesity and cognitive impairment could be attributed to the vascular damage caused by excessive adipose tissue and hyperglycaemia, which may be capable of causing a neuronal injury and subsequently neurodegeneration^(36,37)^. It has also been suggested that excessive adipose tissue triggers a low-grade chronic inflammatory process, causing the increased production of proinflammatory mediators, such as cytokines and macrophages, which can cross the blood−brain barrier and cause neuronal damage^(38,39)^. On the other hand, some studies indicate that underweight older adults are at higher risk for cognitive impairment than those who are overweight^(22)^. Epidemiological studies have also reported that an annual reduction of 1 kg/m^2^ in BMI increases the risk of developing cognitive impairment by 8 %^(40)^. These findings suggest that weight loss and a low BMI could be partially related to brain pathology^(22)^, consistent with the findings of the present study. Our findings also suggest that the association between BMI and cognitive impairment was independent of central adiposity, measured by WC, but the association of WC with cognitive impairment was partially independent of BMI, as the association of cognitive impairment with the lowest quartile of WC disappeared but the magnitude of the association for the highest quartile increased. These results disagree with previous studies which have reported that central obesity is a stronger, and independent, risk factor for cognitive decline and dementia^(18)^. This discrepancy may be explained as BMI is not only a marker of adiposity but also a marker of frailty (loss of muscle mass, especially in underweight individuals); therefore, adjusting for WC would only account for some of the variance of the association between BMI and cognitive impairment. However, accounting for BMI on the association of WC with cognitive impairment may capture most of the variance related to adiposity and its effect on cognitive function.

Although our study reported that underweight and obesity are associated with cognitive impairment, this association may also be bidirectional, where cognitive decline could also lead to changes in adiposity levels. Recent evidence suggests that behavioural changes, such as physical activity, occur 10 years before neurodegenerative diseases are diagnosed^(41)^. This implies that cognitive decline or impairment may lead to obesity or being underweight, as people with cognitive decline are more likely to have limited independence and therefore lower levels of physical activity^(42,43)^. Moreover, other lifestyle behaviours such as poor-quality diet due to individuals with cognitive impairment incapable of performing essential daily living tasks (i.e. eating, shopping, cooking, etc.) may also lead to underweight or obesity^(44)^. There are also other health-related illnesses that may mediate or moderate this association between obesity and cognitive impairment, such as CVD, as individuals with stroke, CHD or diabetes have a higher risk of cognitive impairment which could be explained by adiposity levels or differences in lifestyle behaviours^(41,43–46)^. However, unfortunately due to the cross-sectional nature of the present study we cannot elucidate the underlying roles of existing illness or the bidirectional association between cognitive impairment and adiposity.

### Limitations of this study

It is also important to consider the use of the MMSE, which, although validated and widely used in the Chilean population^(24,25)^, has demonstrated relatively low discriminative power for dementia and should not be considered a diagnostic test^(47)^. Another limitation is that the variables associated with lifestyle, such as physical activity and time spent sitting, were self-reported and could therefore be subject to measurement errors, mainly related to the overestimation of healthy behaviours and, importantly, may be affected by the cognitive ability of the participants^(48)^. Moreover, adiposity was measured using BMI and WC instead of more accurate measurements like dual-energy X-ray absorptiometry. Lastly, as with all cross-sectional and observational studies, causality cannot be confirmed due to the possibility of reverse causation and residual confounding.

### Conclusion

The present study corroborates that both high and low adiposity levels are associated with an increased risk of cognitive impairment in older adults in Chile. These results are of great interest in terms of public health when it is considered that a third of cases of cognitive impairment in the elderly population are attributable to modifiable factors and are therefore potentially preventable if appropriate measures are taken^(49)^.
